# 

**Published:** 1989-05

**Authors:** 


Copyright © Clinical Press Ltd 1989.

**Contents**

31 **Editorial**
33 **The Avon Breast Screening Programme**
Dr Zoe Davies
35 **Practical Aspects of Mammography**
Dr Caroline Boggis
42 **Magnetic Resonance Imaging and Carcinoma of the Breast**
Dr P. G. Cook and Dr P. Goddard
43 **Preoperative Localisation of Impalpable Breast Lesions**
Dr J. F. Reidy
47 **Conservative Surgery for Early Breast Cancer**
Mr R. E. May, Mr D. A. Gillatt, Dr Sally Goodman, Dr Elizabeth Whipp, Dr R. Laing
51 **Epidermal Growth Factor Receptors and Breast Cancer**
Professor J. R. Farndon, Mr S. Nicholson, Mr J. R. C. Sainsbury, Professor A. L. Harris
53 **Breast Cancer and the Radiation Oncologist**
Dr Sally Goodman
55 **Artificial Urinary Sphincters—Early experience**
Dr Sarah Creighton, Mr Paul Abrams
59 **The Conseal Continent Colostomy Appliance**
Sister Jill Downs, Mr David Leaper
50 **Technical Data** from Agfa
54 **Technical Data** from Philips
**Book Reviews**
50 **Essential Paediatric Dermatology**—Julian Verbov
60 **Rheumatic Diseases and the Heart**—John A. Cosh and John V. Lever

